# Supplementary material for: Top-down control of visual cortex by the frontal eye fields through oscillatory realignment
Source: Nat Commun. 2021 Mar 19;12:1757. doi: 10.1038/s41467-021-21979-7 (PMC7979788; doi:10.1038/s41467-021-21979-7)
Supplement: Supplementary file 1 — Supplementary Information [file 41467_2021_21979_MOESM1_ESM.pdf]

Top-down control of visual cortex by the frontal eye fields through oscillatory realignment

Supplementary figures

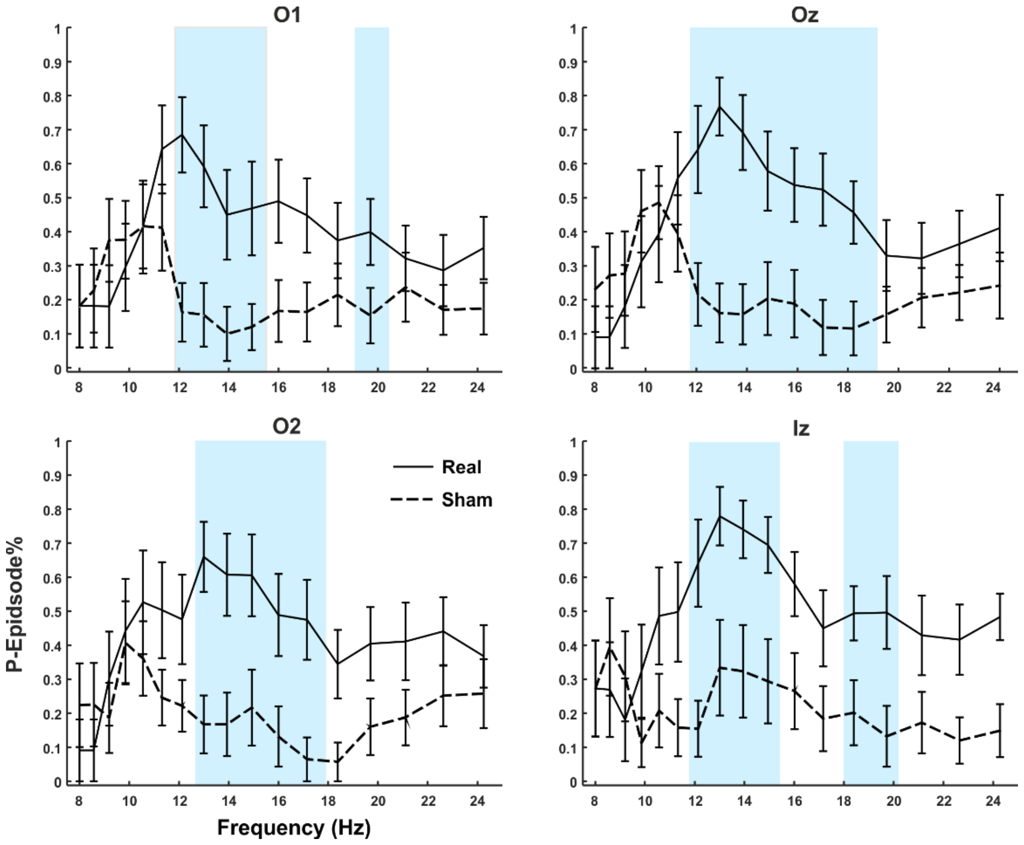

Supplementary figure 1. P-episode - Proportion of time occupied by oscillatory activity as detected by the BOSC method. P-Episode as a function of frequency for each occipital electrode of interest (as in Fig. 1 in the main text) for Real and sham TMS. The time-window used for the analysis is 0-300ms from the TMS pulse. Significant differences between real FEF and sham TMS are highlighted in blue (O1: d=0.65; Oz: d=0.63; O2: d=0.75; Iz (12-15Hz): d=0.75; Iz (18-20Hz) d=0.63; error bars: ±sem, n=11 participants). Source data are provided as a Source Data file.

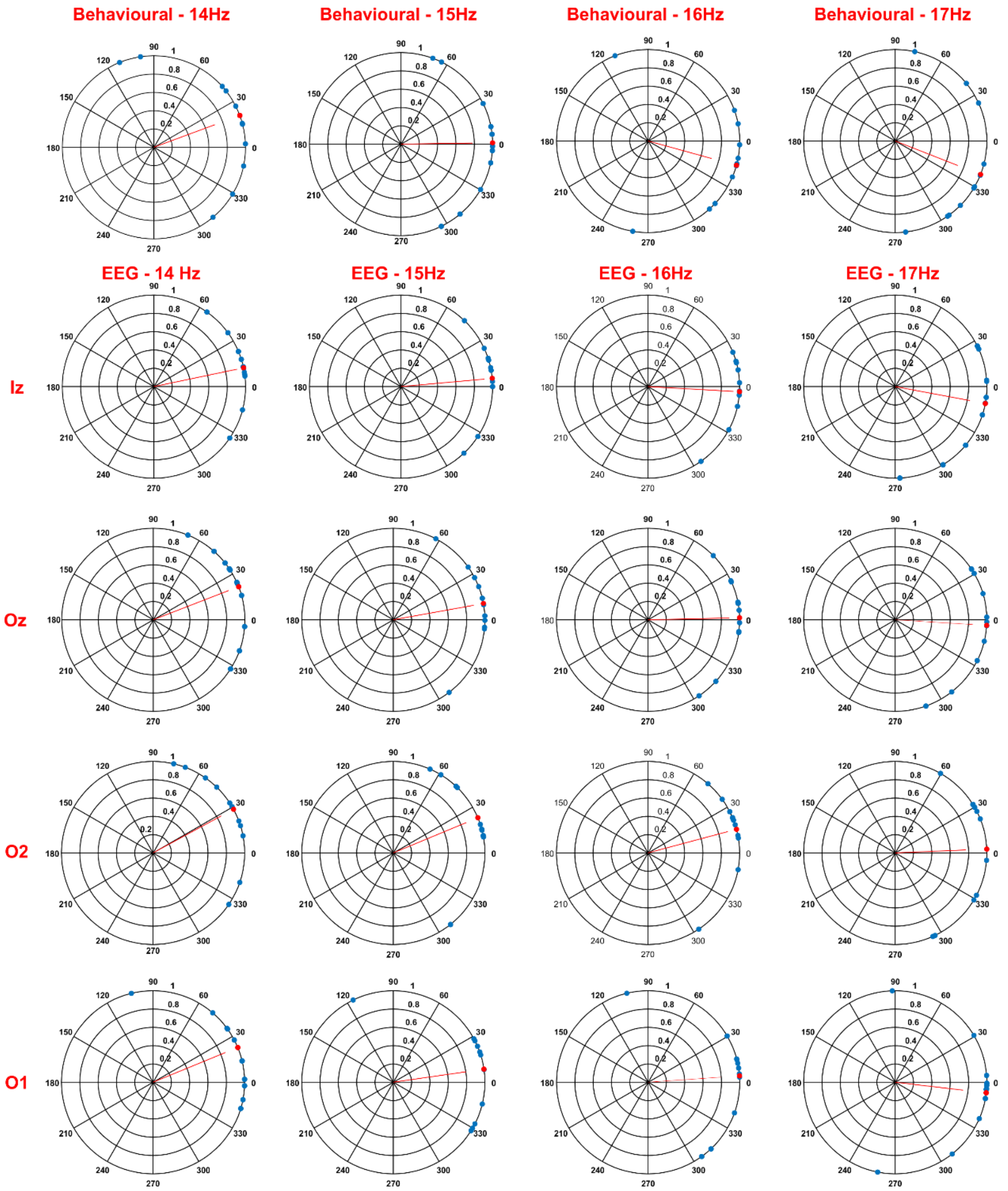

Supplementary figure 2. Phase distribution of behavioral and EEG data. Polar plots indicate the phase distribution across participants (in blue) and the average phase (in red) for which phase of the best fitting cosine function was significantly different from a uniform distribution for the first window (0-200ms) in the motion discrimination task (experiment 2). The corresponding phase from the EEG signal extracted from the occipital electrodes is also shown for comparison. To test for a correlation, we used the circular correlation coefficient rho and the associate p-value as implemented in the circular statistics toolbox for Matlab. For the electrode Iz, a significant phase correlation was found between EEG and behavioral data across participants at 15Hz (rho=.81; p= 0.04, n=11). Source data are provided as a Source Data file.

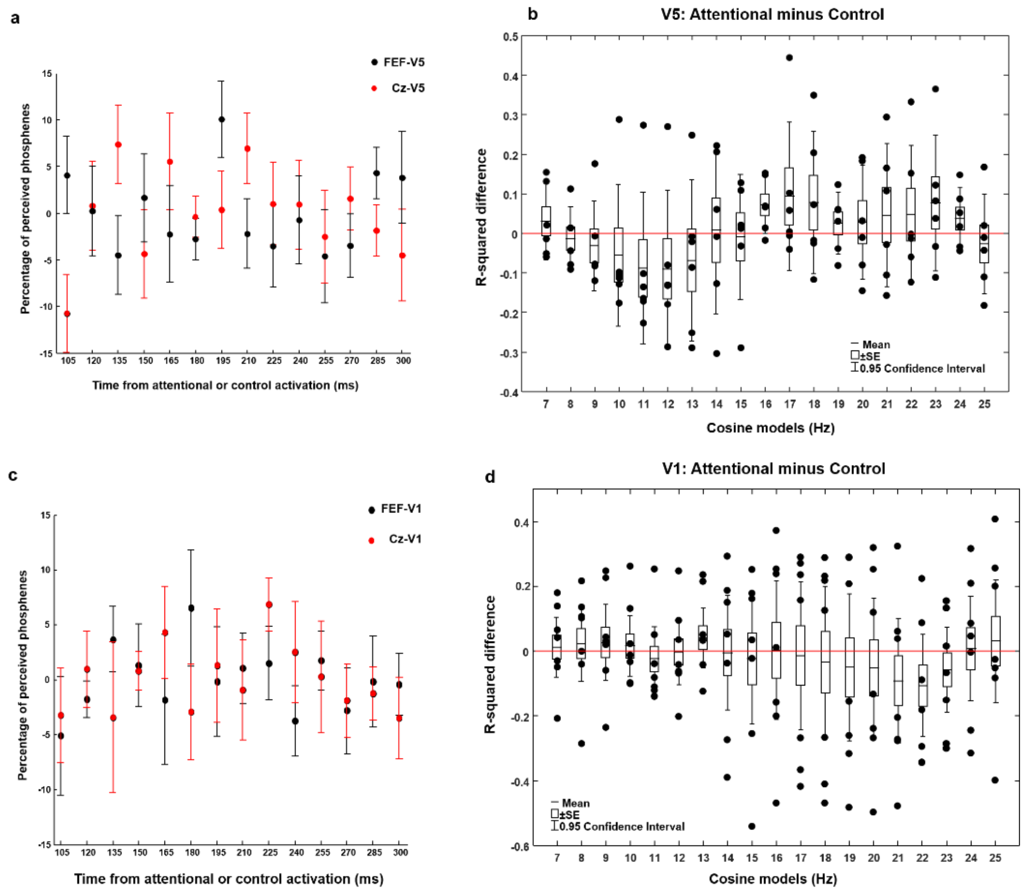

Supplementary figure 3. Effects of FEF-activation on visual cortex (V5 and V1) over time. Phosphene perception for the second time window (105-300 ms from the first pulse delivery) for experiment 3 (a-b) and experiment 4(c-d). **a** Percentage of perceived phosphenes for the attentional (black dots) and control condition (red dots) for the group receiving V5 stimulation (n=6; error bars: ±sem). **b** The two conditions were not statistically different. To test for significant differences, R-squared labels from the two conditions were permuted 500 times and the difference of the mean calculated at each iteration. The null and real data were then compared, and real data considered significant if they fell above the 97.5th percentile. The real difference between conditions did not meet this criterion for any cosine model. In addition, we performed a repeated measure ANOVA with factors Condition (2 levels) and Frequency (19 levels). The Condition x Frequency interaction ($F(18,90)= 0.952$, $p=0.521$) was not significant. Panels c and d show the same information for the group receiving V1 stimulation (n=7; error bars: ±sem). **d** We found no difference between conditions as tested by the same permutation procedure described in **b.** An additional repeated measure ANOVA showed no significant Condition x Frequency interaction ($F_{(18,108)}= 0.373$; $p=0.990$)). Boxplots in **b** and **d**: bounds indicate ±sem, the whiskers represent ±0.95 confidence interval, the horizontal black line the mean R-squared values. Black dots represent single-subject data. Source data are provided as a Source Data file.

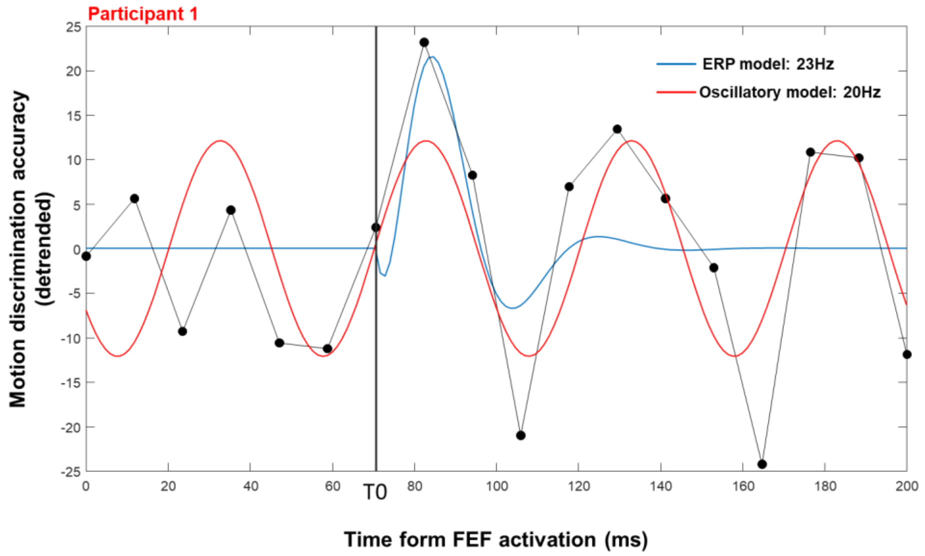

Supplementary figure 4. Cosine and ERP model comparison. Examples of best oscillatory (cosine) and non-oscillatory (ERP) model fits in a representative participant (participant 1). The blue line represents the best ERP model for this participant, the red line the best cosine model (lowest AIC values).
